# Predictors of At-Home Death for Cancer Patients in Rural Clinics in Japan

**DOI:** 10.3390/ijerph182312703

**Published:** 2021-12-02

**Authors:** Jun Watanabe, Hiroyuki Teraura, Kenichi Komatsu, Hironori Yamaguchi, Kazuhiko Kotani

**Affiliations:** 1 Division of Community and Family Medicine, Jichi Medical University, Shimotsuke 329-0498, Japan; m06105jw@jichi.ac.jp (J.W.); m05062ht@jichi.ac.jp (H.T.); komaken@jichi.ac.jp (K.K.); 2 Department of Clinical Oncology, Jichi Medical University, Shimotsuke 329-0498, Japan; yamaguchi@jichi.ac.jp

**Keywords:** critical path, home care, interdisciplinary collaboration, neoplasms, palliative care

## Abstract

Background: The prediction of at-home deaths has become an important topic in rural areas of Japan with an advanced aging society. However, there are no well-established predictors to explain how these factors influence intention. This study aims to investigate the possible predictors of at-home death for cancer patients in rural clinics in Japan. Methods: This is a nationwide cross-sectional survey. A self-administered questionnaire was sent to 493 rural clinics in Japan. The main outcome was the realization of at-home deaths for cancer patients. Results: Among the 264 clinics (54%) that responded to the survey, there were 194 clinics with the realization of at-home death. The use of a clinical pathway (adjusted odds ratio 4.19; 95% confidence interval 1.57–11.19) and the provision of organized palliative care (adjusted odds ratio 19.16; 95% confidence interval 7.56–48.52) were associated with the prediction of at-home death, irrespective of island geography or the number of doctors and nurses. Conclusions: Having a clinical pathway and systematizing palliative care could be important to determine the possibility of at-home deaths for cancer patients in rural clinics in Japan.

## 1. Introduction

Cancer is the leading cause of death worldwide [1]. The Japanese government approved the Cancer Control Act in 2007 and has launched a Basic Plan in the Promote Cancer Control in order to achieve equal cancer treatment and palliative care throughout the country [2]. Despite the increasing number of at-home death as the population ages [3], a minority of deaths take place at home in the real-world [4,5]. As a general trend, palliative care instruction, the presence of clinical pathways, and the provision of at-home palliative care services can increase the chance of at-home death for cancer patients [6,7,8,9,10,11,12,13].

With the advanced aging of the population, the realization of at-home death has become an urgent issue, and the Cancer Control Act [2] has also been applied to rural areas, which are an advanced aging society, in Japan. However, rural areas are underserved both in terms of human resources and medical facilities, and it can be difficult for individuals to access cancer-specialized facilities [14]. As the number of hospitals is limited, clinics have roles in cancer treatment and palliative care in rural areas. Were the factors related to at-home death for cancer patients in rural areas similar to the above-mentioned general trends [6,7,8,9,10,11,12,13] in relation to the realization of at-home death for cancer patients? Since this remains unknown, the aim of the present study was to investigate the possible predictors of at-home death for cancer patients in rural clinics in Japan.

## 2. Materials and Methods

This nationwide cross-sectional survey was conducted using a self-administered questionnaire in 2013. The survey investigated 493 rural clinics in Japan, and the questionnaires were delivered by mail. The clinics were constructed under Japanese law in rural areas, including remote islands, where the population is declining and transportation is inconvenient [15,16]. The clinics were selected randomly considering the whole balance of survey places (i.e., avoidance of sampling from specific places). The questionnaire included the following data: the number of doctors and nurses, the use of a clinical pathway, the provision of organized palliative care, and their experience of at-home death (Appendix A). The main outcome of the present study was the realization of at-home death. The responding clinic’s name was anonymized on the questionnaire form. This study was approved by the Institutional Ethics Committee.

The difference between groups was examined by chi-square test for categorical variables or the Mann–Whitney U-test for continuous variables. A multiple logistic regression model, adjusted with all examined factors, was used to identify the impact of factors on the outcome. *P*-values of < 0.05 were considered to indicate statistical significance.

## 3. Results

Of the 493 rural clinics, 264 (54%) clinics completed the survey. A total of 194 clinics (73%) reported experiencing at-home death in cancer patients. The characteristics of rural clinics with and without the realization of at-home death are listed (Table 1). Rural clinics with the realization of at-home death had more use of a clinical pathway (*p* < 0.01) and more provision of organized palliative care (*p* < 0.01) in comparison to clinics without the realization of at-home death.

Table 2 show the odds ratio (OR) for the realization of at-home death, as analyzed by a logistic regression model. The use of a clinical pathway (adjusted OR 4.19; 95% confidence interval 1.57–11.19; *p* < 0.01) and the provision of organized palliative care (adjusted OR 19.16; 95% confidence interval 7.56–48.52; *p* < 0.01) were positively associated with the realization of at-home death, irrespective of island geography or the number of doctors and nurses.

## 4. Discussion

The present study demonstrated that a clinical pathway and organized palliative care showed a positive independent association with the realization of at-home death for cancer patients in rural clinics of Japan. The findings from this nationwide survey would be useful information for promoting cancer practice in rural areas in Japan.

In the present survey, island geography or the number of doctors and nurses were the insignificant factors in the realization of at-home death. The results might be unexpected because it is typically thought to be difficult to create clinical pathways or receive special instruction in small rural clinics or islands. However, due to the various factors influencing at-home death (e.g., participation in home care by caregivers and family members, the arrangement of community-based informal services), it may cover geographic disadvantages and shortfalls in medical staff within rural areas.

The use of a clinical pathway has been shown to be a general trend in the realization of at-home death [7,11]. This was supported by the results in the present study on rural clinics. The present survey did not detail the contents of the clinical pathways in each clinic; however, since it occasionally seems difficult for the small clinics to create clinical pathways by themselves, some rural clinics could be involved in the clinical pathway of related surrounding hospitals. Involving rural clinics in clinical pathways, which provides access to nearby hospitals with more experience in cancer treatment and palliative care, can be a strategy for the realization of at-home death in rural areas.

Furthermore, systematically organized palliative care has been shown to be a general trend in the realization of at-home death [6,8,9,10,12,13]. This was also supported by the results of the present study in rural clinics. The organization of palliative care includes education or training in care and the existence of interdisciplinary collaboration. Knowledge and skills in palliative care (i.e., the management of pain with opioids and other symptoms related to cancer) is reported to be important for rural physicians to perform palliative care [6,8,9,10,12,13]. This would be similarly important for rural nurses [12]. Using recent technology to overcome the remoteness (i.e., e-learning based on information and communication technology) of rural clinics can be an important strategy for facilitating at-home death [17]. In addition, the usefulness of interdisciplinary work on palliative care has been well established [18,19,20]. Even while there are few human resources, the formation of such a collaborative team would be necessary for rural clinics.

The current study had the advantage of being the first of its kind, and was conducted nationwide with notable findings. Despite this, the present study was associated with some limitations. First, this was not an exhaustive survey, and the response rate was limited. Second, the survey used a self-administered questionnaire. Furthermore, although the organization of palliative care might differ to some degree between the clinics, such a perception was not detailed in the questionnaire. Third, this was a cross-sectional design, which could not determine causality. Fourth, it was performed in 2013, even though the actual status of home care in rural areas might not have changed in recent years. Further studies must be undertaken to address these limitations.

## 5. Conclusions

Having a clinical pathway and systematizing palliative care could be important for the realization of at-home death for cancer patients in rural clinics in Japan. Further studies are warranted.

## Figures and Tables

**Table 1:** The characteristics of the rural clinics studied.

Factors	Realization of At-Home Death (*n* = 194)	Non-Realization of At-Home Death (*n* = 70)	*p*
Island geography	27 (28.7%)	9 (12.9%)	0.83
Multiple doctors	34 (17.5%)	13 (18.6%)	0.85
Number of nurses	3 (2–4)	3 (2–5)	0.91
Use of clinical pathway	59 (30.4%)	6 (8.6%)	<0.01
Organized palliative care	121 (62.4%)	6 (8.6%)	<0.01

Data are presented as the number (percentage) or median (interquartile range). *p*-values were determined by chi-squared test or Mann–Whitney U-test.

**Table 2:** Factors associated with the realization of at-home death.

Factors	Crude OR (95% CI)	*p*	Adjusted OR (95% CI)	*p*
Island geography	1.10 (0.49–2.46)	0.08	1.27 (0.51–3.16)	0.61
Multiple doctors	0.93 (0.46–1.89)	0.85	0.55 (0.19–1.58)	0.27
Number of nurses	0.99 (0.92–1.08)	0.91	0.97 (0.86–1.09)	0.56
Use of clinical pathway	4.66 (1.91–11.40)	<0.01	4.19 (1.57–11.19)	<0.01
Organized palliative care	17.7 (7.29–42.87)	<0.01	19.16 (7.56–48.5)	<0.01

OR, odds ratio; CI, confidence interval. *p*-value were determined by a logistic regression analysis; the adjusted OR was calculated with adjustment for all examined factors.

## Data Availability

The data presented in this study are available on reasonable request and via the ethics committee judgement from the corresponding author.

## Appendix A

The questionnaire survey concerned the following items:

How would you describe the location ofyour facility? -i.e., island, others.

How many doctors are there in your facility?

How many nurses are there in your facility?

Does your facility use any clinical pathway for cancer care? -Yes or No.

Does your facility provide organized palliative care? -Yes or No.

Was at-home death realized in your facility in the present situation? -Yes or No (the realization was not acceptable and/or the hospital admission was recommended).
